# Alcohol use among Croatian adolescents: the alignment of 13-year-old and 15-year-old girls with boys, and the impact of the COVID-19 pandemic

**DOI:** 10.3325/cmj.2024.65.483

**Published:** 2024-12

**Authors:** Maja Valentić, Tonka Karin, Luka Šimetin, Lara Petković, Filip Šimetin, Mirjana Kujundžić Tiljak

**Affiliations:** 1 Croatian Institute of Public Health, Zagreb, Croatia; 2 University of Zagreb School of Medicine, Zagreb, Croatia; 3 Department of Medical Statistics, Epidemiology and Medical Informatics, Andrija Štampar School of Public Health, University of Zagreb School of Medicine, Zagreb, Croatia

## Abstract

**Aim:**

To determine age and gender patterns of alcohol use among Croatian pupils and assess whether alcohol use was associated with factors related to school, peers, family, and the COVID-19 pandemic.

**Methods:**

Data were collected from the 2022 Health Behavior in School-aged Children cross-sectional study conducted in Croatia involving 5338 pupils. Pearson χ^2^ test and multivariate logistic regression were performed.

**Results:**

At the age of 11, boys were drinking alcohol more than girls (*P* < 0.001), while 13- and 15-year-old girls aligned with boys. Lifetime alcohol use was positively associated with schoolwork pressure in 11-year-old girls (OR 3.28, CI 1.36-7.75) and boys (OR 1.87, CI 1.03-3.37). The COVID-19 pandemic negatively affected mental health in 13- (OR 2.21, 1.56-3.13) and 15-year-old girls (OR 1.50, CI 1.01-2.23), and life in 15-year-old boys (OR 1.83, 1.03-3.27). Recent alcohol use was positively associated with hospitalization of a close family member for COVID-19 in 11-year-old girls (OR 2.35, 1.05-5.28), low peer support in 13-year-old boys (OR 1.49, 1.01-2.20), difficult communication with father in 15-year-old girls (OR 1.49,1.05-2.12), negative COVID-19 impact on mental health in 13-year-old girls (OR 1.67,1.13-2.47), and negative COVID-19 impact on life in 15-year-old boys (OR 1.79, 1.08-2.98). Lifetime drunkenness was positively associated with negative COVID-19 impact on mental health in 13- (OR, 2.03,1.28-3.21) and 15-year-old girls (OR 2.12, 1.49-3.01), and with positive or neutral COVID-19 impact on life in 15-year-old girls (OR 0.65, 0.43-0.97).

**Conclusion:**

Preventive activities should offer support systems to minimize the negative COVID-19 impact, with special attention to girls' needs.

The high proportion of adolescents engaging in cigarettes and alcohol use is considered a critical public health issue (1). Substance use is typically initiated in adolescence, with alcohol being the most used substance (2). Alcohol consumption at that age is related to many adverse outcomes. While boys are more prone to drinking than girls, the difference is becoming less and less apparent (3).

The impact of various school, peer, and family factors on adolescent alcohol use has been thoroughly researched (4-27). However, there is limited research on the association of schoolwork pressure, peer support, and parental communication with adolescent alcohol use (5,10,12,13,18,23,27). Research results are varying depending on the tools used to assess these measures, interactions with other indicators, and the overall context of the study (5,10,12,13,18,23,27).

A Dutch study found no association between schoolwork pressure and alcohol use in the last 30 days in 15- and 16-year-old pupils (18). A study across 37 countries showed that increased schoolwork pressure in 15-year-old girls was associated with a decline in alcohol use (27). On the other hand, a Welsh study demonstrated that schoolwork pressure was a risk factor for frequent binge drinking in pupils aged 11-15 (23).

When it comes to peer support, the Welsh study (23) found that peer support was a risk factor for frequent binge drinking in 11-15-year-old adolescents, whereas a US study in 15-year-olds (12), and a Belgian study in 10- to 14-year-olds (5) showed no association between peer support and alcohol use in the last 30 days for both genders.

Regarding parental communication, a Portuguese study (13) showed a positive association between alcohol use and difficult communication with mother for boys attending the sixth, eighth, and tenth grades, whereas difficult communication with parents was one of the predictors of casual drinking and binge drinking for Israeli adolescents aged 11-17 (10). In a Welsh study, easy communication with parents was a protective factor against frequent binge drinking in pupils aged 11-15 (23).

The COVID-19 pandemic significantly affected billions of people worldwide, including adolescents, who had to adapt to social distancing rules, temporary school closures, and limited leisure activities (28). Experts raised concern about a potential impact of these measures on adolescents’ mental well-being (29). While research on the consequences of the pandemic on children's and adolescents’ well-being and lifestyle is still scarce, such an experience can significantly affect their lives, especially in the area of health-related behaviors (29,30).

It is still unclear whether adolescent alcohol use increased or decreased during the COVID-19 pandemic (28,30-40). Some studies indicated an increase in alcohol consumption, while others suggested a decrease or no change (28,30-40).

Among the factors that could have contributed to more excessive adolescent drinking during the COVID-19 pandemic are isolation from friends and peers, boredom, stress, anxiety and depression, uncertainty about school closures, changes in routine activities, lack of physical space at home, health complications, and financial worsening in families (28-35,37,40). On the other hand, lack of parties and physical contact with friends, harder access to alcohol, and higher parental monitoring could have led to a reduction in alcohol use (28,30,31).

Croatia has a high prevalence of alcohol consumption among pupils, but it no longer ranks first among the countries involved in Health Behavior in School-aged Children (HBSC) study (3). The proportion of pupils who got drunk in their lifetime increased with age in both boys and girls (3), whereas boys in all age groups were more prone to drinking than girls (3).

This study aimed to determine the patterns of alcohol use of Croatian pupils by gender and age in the spring of 2022, two years after the beginning of the COVID-19 pandemic. Additionally, we aimed to investigate the association of alcohol use patterns with schoolwork pressure, peer support, communication with mother and father, hospitalization of a close family member for COVID-19, and COVID-19 impact on mental health and life as a whole.

## Methods

### Sample

The data were obtained from the 2022 HBSC study conducted in Croatia. The HBSC study is a World Health Organozation cross-sectional study conducted every four years across countries in Europe and North America (7,41). It gathers data from 11-, 13-, and 15-year-old pupils using an international standardized protocol (7,41).

The Croatian sample, based on the official list of schools released by the Ministry of Science and Education, was obtained by using the school class as a sampling unit (41). School classes were randomly selected at the national level and, in the case of 15-year-olds, stratified by the type of high school (8,41). The data were collected from pupils attending the fifth year and seventh year of elementary school, and those attending the first year of high school (aged 11, 13, and 15 years, respectively). Additionally, pupils outside these specific age ranges but still enrolled in the selected classes were included in the study, following the emphasis on class-based sampling. There were 5338 pupils in the sample, of which 51.69% were girls (2759), with a response rate of 64.6%. The sample included 1763 pupils aged 11 (average age 11.07), 1940 pupils aged 13 (average age 12.96), and 1635 pupils aged 15 (average age 14.99).

An international standardized questionnaire was used as a research instrument, after the process of translation into Croatian and back-translation (7,41). The data collection took place between March and May 2022. The survey was conducted anonymously and voluntarily, with passive parental consent. The online questionnaire was self-administered by pupils in the classroom under the supervision of a teacher, using the online platform LimeSurvey.

### Measures

Three measures of alcohol use were employed as dependent variables. Lifetime alcohol use was assessed with the question, “On how many days (if any) have you drunk alcohol in a lifetime?”. The response options on a seven-point scale ranged from “never” to “30 days (or more)”. Respondents who had never drunk alcohol were compared with those who had drunk alcohol on one or more days in a lifetime.

Recent alcohol use was assessed with the question, “On how many days (if any) have you drunk alcohol in the last 30 days?”, with the same response options as for lifetime alcohol use. Respondents who had not drunk in the last 30 days were compared with those who had drunk alcohol on one or more days in the last 30 days.

Lifetime drunkenness was evaluated with the question, “Have you ever in your lifetime had so much alcohol that you were really drunk?” with five-answer options ranging from “no, never” to “yes, more than 10 times.” Respondents were classified as never having been drunk vs having been drunk one or more times in a lifetime.

Schoolwork pressure, peer support, communication with mother and father, hospitalization of a close family member for COVID-19, COVID-19 impact on mental health, and COVID-19 impact on life as a whole were used as independent variables.

Schoolwork pressure was evaluated with the question, “How pressured do you feel by the schoolwork you have to do?” Response categories on a four-point scale were recoded as no pressure and certain pressure.

Peer support was measured using The Multidimensional Scale of Perceived Social Support (42) with the following items: “My friends really try to help me,” “I can count on my friends when things go wrong,” “I have friends with whom I can share my joys and sorrows,” and “I can talk about my problems with my friends.” Response options ranged from 1 or “very strongly disagree” to 7 or “very strongly agree.” The four-item scores were summed and divided by the number of items (ie, four). The overall scores were recoded into two categories: low peer support (score below 5.5) vs high peer support (score 5.5 and above).

Communication with mother and father was measured using the question, “How easy is it for you to talk to the following persons about things that really bother you?” separately for each parent. Four-answer options were dichotomized as easy communication with mother vs difficult communication with mother, and the same was applied to communication with father.

Dichotomized categories for the question “Was anyone in your close family (ie, parent, sibling, or grandparent) treated in hospital for COVID-19?” were “yes” and “no.”

Regarding the questions on the impact of the COVID-19 pandemic on mental health and life as a whole, responses on a five-point Likert scale were recoded as a negative COVID-19 impact and a positive or neutral COVID-19 impact.

### Statistical analysis

Descriptive statistics was used to summarize the data. Gender and age differences in alcohol use were tested by using Pearson χ^2^ test. Multivariate logistic regression was performed separately by gender and age groups (11, 13, and 15 years) for dependent and independent dichotomized variables. The results of logistic regression are presented as odds ratios (OR) with 95% confidence intervals (CI). The statistical significance level was set at *P* < 0.05. Statistical analysis was conducted with SPSS, version 28 (IBM Corp., Armonk, NY, USA).

## Results

### Sample

More than half of the respondents had never drunk alcohol (56.6%), while 43.4% had drunk alcohol at least once in their lifetime. Many of them had not drunk alcohol recently (73.2%), nor had they ever been drunk (78.7%). The majority of respondents (86.9%) experienced a certain level of pressure from their schoolwork. Many respondents felt they had high peer support (62%), as well as easy communication with their mother (84.3%) and father (74.2%). Hospitalization of a close family member for COVID-19 was reported by 14.2% of respondents.

More than half of all respondents reported a positive or neutral impact of COVID-19 on mental health (57.7%), while 61.9% reported a positive or neutral impact of COVID-19 on life as a whole (Table 1).

**Table 1:** Characteristics of Croatian pupils involved in the research

Variable	N	%
**Gender**		
male	2579	48.3
female	2759	51.7
**Age**		
11	1763	33
13	1940	36.4
15	1635	30.6
**Lifetime alcohol use**		
no	2879	56.6
yes	2212	43.4
**Recent alcohol use**		
no	3751	73.2
yes	1373	26.8
**Lifetime drunkenness**		
no	4038	78.7
yes	1095	21.3
**Schoolwork pressure**		
no	666	13.1
yes	4415	86.9
**Peer support**		
high peer support	3052	62
low peer support	1867	38
**Communication with mother**		
easy	3952	84.3
difficult	736	15.7
**Communication with father**		
easy	3376	74.2
difficult	1176	25.8
**Hospitalization of a close family member for COVID-19**		
no	4038	85.8
yes	668	14.2
**COVID-19 impact on mental health**		
Positive or neutral	2906	57.7
Negative	2134	42.3
**COVID-19 impact on life as a whole**		
positive or neutral	3134	61.9
negative	1932	38.1

At age 11, lifetime alcohol use was higher in boys than in girls (23.4% vs 15.0%). However, girls had higher percentages of lifetime alcohol use at ages 13 (45.8% vs 41.7%) and 15 (71.5% vs 67.4%). Regarding recent alcohol use, the percentage was higher for 11-year-old boys compared with girls (10.7 vs 5.5), but the opposite was observed at ages 13 (24.8% vs 21.5%) and 15 (51.9% vs 50.8%). A higher percentage of boys report lifetime drunkenness compared with girls at ages 11 (9.3% vs 3.8%) and 13 (17.2% vs 15.6%), but the opposite was true at the age of 15 (43.7% vs 42.8%) (Table 2).

**Table 2:** Frequency distribution of the dependent variables by age and gender

Age (years)	Variable	Boys		Girls		χ2 value (df = 1)	*P* value
		N	%	N	%		
**11**	**Lifetime alcohol use**					19.021	<0.001
	no	640	76.6	730	85		
	yes	195	23.4	129	15		
	**Recent alcohol use**					15.685	<0.001
	no	752	89.3	813	94.5		
	yes	90	10.7	47	5.5		
	**Lifetime drunkenness**					20.917	<0.001
	no	769	90.7	827	96.2		
	yes	79	9.3	33	3.8		
**13**	**Lifetime alcohol use**					3.277	0.070
	no	528	58.3	508	54.2		
	yes	377	41.7	430	45.8		
	**Recent alcohol use**					2.939	0.086
	no	713	78.5	708	75.2		
	yes	195	21.5	234	24.8		
	**Lifetime drunkenness**					0.875	0.350
	no	756	82.8	796	84.4		
	yes	157	17.2	147	15.6		
**15**	**Lifetime alcohol use**					3.103	0.078
	no	232	32.6	240	28.5		
	yes	479	67.4	602	71.5		
	**Recent alcohol use**					0.196	0.658
	no	355	49.2	409	48.1		
	yes	366	50.8	441	51.9		
	**Lifetime drunkenness**					0.137	0.711
	no	409	57.2	480	56.3		
	yes	306	42.8	373	43.7		

Statistically significant gender differences were found among 11-year-olds in lifetime alcohol use (χ^2^ = 19.021, *P* < 0.001), recent alcohol use (χ^2^ = 15.685, *P* < 0.001), and lifetime drunkenness (χ^2^ = 20.917, *P* < 0.001), with boys consuming alcohol more than girls (Table 2).

### Lifetime alcohol use

Multivariate binary logistic regression, performed separately by gender and age groups, showed higher odds of lifetime alcohol use in 11-year-old girls (OR 3.28, CI 1.36-7.75) and boys (OR 1.87, CI 1.03-3.37) who felt pressured by schoolwork than in those who did not feel pressured by schoolwork. Thirteen-year-old girls whose mental health was negatively affected by COVID-19 showed higher odds of lifetime alcohol use (OR 2.21, CI 1.56-3.13) than those whose mental health was positively or neutrally affected by COVID-19. At age 15, boys with a negative COVID-19 impact on life as a whole showed higher odds of lifetime alcohol use (OR 1.83, CI 1.03-3.27) than those with a positive or neutral COVID-19 impact on their lives. Similarly, girls of the same age with a negative COVID-19 impact on mental health showed higher odds of lifetime alcohol use (OR 1.50, CI 1.01-2.23) than those with a positive or neutral impact of COVID-19 on their mental health (Table 3).

**Table 3:** Association of factors related to school, peer, family, and COVID-19 with lifetime alcohol use (multivariate binary logistic regression)

Age (years)	School, peer, family, and COVID-19 factors	Lifetime alcohol use	
		Boys	Girls
		OR (95% CI); p	OR (95% CI); p
11	Certain schoolwork pressure vs no schoolwork pressure	1.87 (1.03-3.37); 0.039	3.28 (1.39-7.75); 0.007
	Low peer support vs high peer support	1.31 (0.87-1.99); 0.200	1.26 (0.77-2.07); 0.353
	Difficult vs easy communication with mother	1.16 (0.74-3.53); 0.230	1.01 (0.47-2.17); 0.985
	Difficult vs easy communication with father	1.14 (0.60-2.17); 0.685	1.59 (0.90-2.80); 0.108
	Hospitalization of a close family member for COVID-19 vs no hospitalization	1.19 (0.64-2.21); 0.589	1.60 (0.88-2.90); 0.124
	Negative vs positive COVID-19 impact on mental health	1.27 (0.73-2.20); 0.398	1.23 (0.67-2.23); 0.508
	Negative vs positive COVID-19 impact on life as a whole	1.28 (0.77-2.12); 0.342	0.69 (0.35-1.37); 0.288
13	Certain schoolwork pressure vs no schoolwork pressure	1.12 (0.67-1.89); 0.670	1.07 (0.55-2.09); 0.834
	Low peer support vs high peer support	1.06 (0.78-1.45); 0.710	1.00 (0.72-1.39); 0.985
	Difficult vs easy communication with mother	1.36 (0.82-2.25); 0.229	1.64 (1.07-2.50); 0.229
	Difficult vs easy communication with father	1.12 (0.72-1.74); 0.612	1.26 (0.88-1.79); 0.612
	Hospitalization of a close family member for COVID-19 vs no hospitalization	1.17 (0.72-1.90); 0.520	1.17 (0.77-1.79); 0.462
	Negative vs positive COVID-19 impact on mental health	1.12 (0.72-1.75); 0.621	2.21 (1.56-3.13); <0.001
	Negative vs positive COVID-19 impact on life as a whole	1.32 (0.86-2.03); 0.205	0.92 (0.61-1.38); 0.205
15	Certain schoolwork pressure vs no schoolwork pressure	1.05 (0.67-1.65); 0.835	0.90 (0.49-1.67); 0.736
	Low peer support vs high peer support	0.88 (0.61-1.28); 0.503	0.93 (0.65-1.34); 0.703
	Difficult vs easy communication with mother	1.26 (0.69-2.32); 0.450	1.49 (0.92-2.39); 0.104
	Difficult vs easy communication with father	0.85 (0.50-1.42); 0.527	1.40 (0.95-2.07); 0.091
	Hospitalization of a close family member for COVID-19 vs no hospitalization	1.68 (0.99-2.84); 0.053	0.98 (0.63-1.54); 0.940
	Negative vs positive COVID-19 impact on mental health	0.93 (0.59-1.56); 0.787	1.50 (1.01-2.23); 0.043
	Negative vs positive COVID-19 impact on life as a whole	1.83 (1.03-3.27); 0.041	0.69 (0.44-1.06); 0.087

### Recent alcohol use

In 11-year-old girls, the odds of recent alcohol use were higher for those with close family members hospitalized for COVID-19 (OR 2.35, CI 1.05-5.28) than in those without such hospitalizations. Thirteen-year-old boys with low peer support were more likely to have used alcohol recently (OR 1.49, CI 1.01-2.20) than those with high peer support. Thirteen-year-old girls showed a positive association between recent alcohol use and a negative impact of COVID-19 on mental health (OR 1.67, CI 1.13-2.47) (Table 4).

**Table 4:** Association of factors related to school, peer, family, and COVID-19 with recent alcohol use (multivariate binary logistic regression)

Age (years)	School, peer, family, and COVID-19 factors	Recent alcohol use	
		Boys	Girls
		OR (95% CI); p	OR (95% CI); p
**11**	Certain schoolwork pressure vs no schoolwork pressure	1.65 (0.76-3.60); 0.208	3.34 (0.78-14.28); 0.104
	Low peer support vs high peer support	0.97 (0.55-1.69); 0.905	1.90 (0.94-3.86); 0.075
	Difficult vs easy communication with mother	1.50 (0.57-3.97); 0.416	0.74 (0.24-2.31); 0.600
	Difficult vs easy communication with father	1.20 (0.52-2.76); 0.676	2.10 (0.95-4.62); 0.066
	Hospitalization of a close family member for COVID-19 vs no hospitalization	1.49 (0.69-3.24); 0.311	2.35 (1.05-5.28); 0.038
	Negative vs positive COVID-19 impact on mental health	1.38 (0.68-2.79); 0.370	0.72 (0.27-1.92); 0.505
	Negative vs positive COVID-19 impact on life as a whole	1.19 (0.62-2.31); 0.605	1.15 (0.44-3.01); 0.779
**13**	Certain schoolwork pressure vs no schoolwork pressure	1.01 (0.53-1.93); 0.971	1.48 (0.60-3.61); 0.393
	Low peer support vs high peer support	1.49 (1.01-2.20); 0.044	1.25 (0.87-1.80); 0.236
	Difficult vs easy communication with mother	1.44 (0.81-2.58); 0.219	1.31 (0.83-2.08); 0.251
	Difficult vs easy communication with father	1.07 (0.63-1.81); 0.801	1.28 (0.85-1.92); 0.234
	Hospitalization of a close family member for COVID-19 vs no hospitalization	1.34 (0.76-2.35); 0.316	1.26 (0.79-2.01); 0.338
	Negative vs positive COVID-19 impact on mental health	1.22 (0.72-2.07); 0.461	1.67 (1.13-2.47); 0.010
	Negative vs positive COVID-19 impact on life as a whole	1.06 (0.63-1.79); 0.818	1.09 (0.70-1.69); 0.719
**15**	Certain schoolwork pressure vs no schoolwork pressure	1.49 (0.98-2.28); 0.063	0.76 (0.43-1.34); 0.341
	Low peer support vs high peer support	0.86 (0.60-1.22); 0.390	0.86 (0.62-1.19); 0.371
	Difficult vs easy communication with mother	0.80 (0.46-1.40); 0.428	0.92 (0.61-1.38); 0.668
	Difficult vs easy communication with father	0.93 (0.57-1.52); 0.770	1.49 (1.05-2.12); 0.026
	Hospitalization of a close family member for COVID-19 vs no hospitalization	1.32 (0.83-2.09); 0.242	1.03 (0.69-1.54); 0.896
	Negative vs positive COVID-19 impact on mental health	0.83 (0.51-1.33); 0.429	1.31 (0.92-1.86); 0.129
	Negative vs positive COVID-19 impact on life as a whole	1.79 (1.08-2.98); 0.024	0.69 (0.47-1.02); 0.063

Regarding 15-year-olds, boys whose lives as a whole were negatively affected by COVID-19 were more likely to have used alcohol recently (OR 1.79, CI 1.08-2.98) than those whose lives were positively or neutrally affected by COVID-19. Girls of the same age reporting communication difficulties with their fathers showed higher odds for recent alcohol use (OR 1.49, CI 1.05-2.12) than those who easily communicated with their fathers (Table 4).

### Lifetime drunkenness

Lifetime drunkenness (Table 5) was positively associated with a negative impact of COVID-19 on mental health in 13-year-old (OR 2.03, CI 1.29-3.21) and 15-year-old girls (OR 2.12, CI 1.49-3.01). Fifteen-year-old girls had higher odds of drunkenness in their lifetime if COVID-19 positively or neutrally affected their life as a whole compared with those whose life as a whole was negatively affected by COVID-19 (OR 0.65, CI 0.43-0.97).

**Table 5:** Association of factors related to school, peer, family, and COVID-19 with lifetime drunkenness (multivariate binary logistic regression)

Age (years)	School, peer, family, and COVID-19 factors	Lifetime drunkenness	
		Boys	Girls
		OR (95% CI); p	OR (95% CI); p
11	Certain schoolwork pressure vs no schoolwork pressure	2.21 (0.85-5.77); 0.105	0.70 (0.27-1.83); 0.467
	Low peer support vs high peer support	1.15 (0.63-2.10); 0.654	1.54 (0.65-3.66); 0.327
	Difficult vs easy communication with mother	1.55 (0.51-4.72); 0.442	0.48 (0.09-2.51); 0.386
	Difficult vs easy communication with father	0.54 (0.18-1.60); 0.268	1.51 (0.56-4.12); 0.416
	Hospitalization of a close family member for COVID-19 vs no hospitalization	2.09 (0.97-4.53); 0.062	1.35 (0.45-4.06); 0.597
	Negative vs positive COVID-19 impact on mental health	1.88 (0.80-3.91); 0.094	1.26 (0.41-3.89); 0.684
	Negative vs positive COVID-19 impact on life as a whole	1.43 (0.71-2.87); 0.317	0.00 (0.00-0.00); 0.996
13	Certain schoolwork pressure vs no schoolwork pressure	0.85 (0.44-1.66); 0.641	1.03 (0.39-2.72); 0.960
	Low peer support vs high peer support	1.15 (0.76-1.76); 0.513	0.92 (0.60-1.43); 0.717
	Difficult vs easy communication with mother	1.73 (0.93-3.20); 0.082	1.17 (0.68-1.99); 0.571
	Difficult vs easy communication with father	1.15 (0.65-2.03); 0.629	1.52 (0.95-2.72); 0.082
	Hospitalization of a close family member for COVID-19 vs no hospitalization	0.79 (0.39-1.60); 0.512	1.65 (0.98-2.79); 0.060
	Negative vs positive COVID-19 impact on mental health	1.07 (0.59-1.92); 0.834	2.03 (1.29-3.21); 0.002
	Negative vs positive COVID-19 impact on life as a whole	0.97 (0.54-1.72); 0.907	0.67 (0.38-1.15); 0.144
15	Certain schoolwork pressure vs no schoolwork pressure	1.06 (0.69-1.62); 0.804	0.81 (0.46-1.44); 0.481
	Low peer support vs high peer support	0.88 (0.62-1.25); 0.465	0.93 (0.67-1.30); 0.684
	Difficult vs easy communication with mother	1.13 (0.64-1.98); 0.674	1.33 (0.89-1.99); 0.175
	Difficult vs easy communication with father	0.97 (0.59-1.59); 0.895	1.35 (0.95-1.92); 0.095
	Hospitalization of a close family member for COVID-19 vs no hospitalization	1.34 (0.85-2.11); 0.206	1.08 (0.72-1.64); 0.702
	Negative vs positive COVID-19 impact on mental health	0.80 (0.49-1.28); 0.348	2.12 (1.49-3.01); <0.001
	Negative vs positive COVID-19 impact on life as a whole	1.48 (0.90-2.43); 0.119	0.65 (0.43-0.97); 0.034

The association of dependent variables with other independent variables by the gender and age groups was not found.

## Discussion

In this study, as adolescents got older, both boys and girls had higher percentages of lifetime and recent alcohol use as well as lifetime drunkenness. Previous studies showed higher proportions of alcohol use among boys than girls (3), but this pattern changed during COVID-19 pandemic. At the age of 11, boys were more prone to drinking alcohol than girls. However, at the ages of 13 and 15 girls were aligned with boys, with slightly higher proportions in most alcohol use patterns.

Schoolwork pressure emerged as a risk factor for lifetime alcohol use in 11-year-old boys and girls, which, according to our knowledge, was not previously observed. However, this association became less strong as the adolescents got older, which suggests that school pressure may contribute to early alcohol use, but its impact decreases over time.

In our study, peer support played a role in recent alcohol use in 13-year-old boys. Respondents with low peer support were more likely to have used alcohol recently than those with high peer support. This finding is new, as previous studies observed the opposite or found no association between peer support and adolescent alcohol use (5,12,23).

Difficult communication with father was associated with recent alcohol use in 15-year-old girls. Respondents reporting difficult communication with fathers had higher odds of recent alcohol use than those reporting easy communication. Previous studies have also found associations between difficult communication with parents and alcohol use (10,13,23). These findings emphasize the significance of positive communication between parents and children, especially during mid-adolescence, when substance use is more prevalent.

The impact of COVID-19 on alcohol consumption across all age groups suggests a broader context that goes beyond family, peers, and school influences. Stressors from the COVID-19 pandemic might have led to using alcohol as a coping mechanism (28-35,37,40).

Boys aged 15 whose lives as a whole were negatively affected by the COVID-19 pandemic had higher odds of lifetime alcohol use and recent alcohol use than those whose lives were positively or neutrally affected by COVID-19. In 11-year-old girls, the odds of recent alcohol use were higher for those with close family members hospitalized for COVID-19 than for those without such hospitalizations. Thirteen-year-old girls whose mental health was negatively affected by COVID-19 showed higher odds of lifetime alcohol use, recent alcohol use, and lifetime drunkenness than those with a positive and neutral COVID-19 impact on mental health. Girls aged 15 with a negative COVID-19 impact on mental health were more likely to have used alcohol and got drunk at least once in their lives than those with a positive or neutral COVID-19 impact on mental health.

The strong association between a negative COVID-19 impact on their lives and higher odds of alcohol use for boys might be due to their tendency for assertive and outward behaviors, whereas the link between a negative COVID-19 impact on mental health and alcohol use among girls could be due to their tendency to internalize emotions (43).

In previous studies, the negative impact of COVID-19 was more apparent in girls than in boys (40,44). However, this study showed that COVID-19 also negatively affected boys, leading to higher odds of alcohol use.

Finally, the finding that 15-year-old girls were more likely to experience lifetime drunkenness if their lives were positively or neutrally affected by COVID-19 than those whose lives were negatively affected by COVID-19 warrants further exploration.

Several limitations should be considered when interpreting the findings of this study. Self-report measures of alcohol use and other variables may be subject to reporting bias and memory inaccuracies. The cross-sectional design of the research prevents us from establishing causal relationships between variables. Regarding the recommendations for future research, longitudinal studies could offer even stronger insights into the complex relationship between school, peer, family, and COVID-19-related factors, and alcohol use over some time.

This study highlights the need for effective support systems to reduce harmful coping mechanisms, such as alcohol consumption. Preventive activities and strategies in response to negative COVID-19 impact should be directed toward both genders, with a specific focus on girls’ needs. All these efforts are crucial for creating interventions adapted to the needs of adolescents in a rapidly changing world.
